# Lemierre’s Syndrome Complicated by Descending Mediastinitis

**DOI:** 10.7759/cureus.99740

**Published:** 2025-12-20

**Authors:** Imane Lefqih, Taha Ismail Sefrioui, Fatima Zahra Ammor, El Mehdi Maidi, Mohamed Mehdi El Fakiri

**Affiliations:** 1 Department of Anatomy and Applied Anatomy, Faculty of Medicine and Pharmacy, Ibn Zohr University, Agadir, MAR; 2 Department of Thoracic Surgery, Mohammed VI University Hospital, Agadir, MAR; 3 Department of Otorhinolaryngology - Head and Neck Surgery, Mohammed VI University Hospital, Agadir, MAR; 4 Department of Otorhinolaryngology – Head and Neck Surgery, Mohammed VI University Hospital, Agadir, MAR

**Keywords:** antibiotics, anticoagulant treatment, descending necrotizing mediastinitis, lemierre’s syndrome, multidisciplinary management, surgical treatment

## Abstract

Lemierre’s syndrome (LS) is a rare, potentially life-threatening complication of acute oropharyngeal infection that may progress to mediastinitis if treatment is delayed. We report a case of a 35-year-old woman with no prior medical history who had been using products promoting body mass gain, potentially corticosteroid-based, for three months. She presented with neck swelling, odynophagia, and fever for two weeks and reported New York Heart Association class III dyspnea. Contrast-enhanced cervicothoracic CT revealed laterocervical, parapharyngeal, and tonsillar abscesses extending to the anterior cervical region with air bubbles, a retrosternal collection, and right pericardial and pleural effusions, along with thrombosis of the superior vena cava, retromandibular vein, and external jugular vein. Laboratory analyses showed elevated inflammatory markers and purulent pleural fluid. A diagnosis of LS complicated by descending mediastinitis was made. The patient underwent drainage of the various collections, including thoracic drainage. Her condition improved satisfactorily with surgical treatment, antibiotics, anticoagulation, and local care, and she was subsequently discharged from the hospital. LS is a serious condition that can lead to life-threatening complications such as mediastinitis, requiring rapid, multidisciplinary, and appropriate management.

## Introduction

Lemierre’s syndrome (LS) is a rare and potentially fatal complication of acute oropharyngeal infection and, less commonly, of otitis media or mastoiditis. First described in 1936, it is usually caused by *Fusobacterium necrophorum*, leading to thrombophlebitis of the internal jugular vein and sepsis [1,2]. The infection spreads through the formation of septic emboli to other organs and generally affects young, healthy adults [3,4].

Complications may include pneumonia, empyema, or pulmonary infarction induced by septic emboli. In our case, the patient developed mediastinitis with right pleural empyema [4]. The mortality rate has decreased considerably, reaching 2-4% after the introduction of antibiotics in the 21st century, but it can increase to 30% when complicated by mediastinitis [4,5]. Most cases are caused by *F. necrophorum*, followed by *Fusobacterium nucleatum*, unspecified *Fusobacterium *species, *Streptococcus*, methicillin-resistant *Staphylococcus aureus*, and *S. aureus *[6].

CT is the most commonly used modality to demonstrate venous thrombophlebitis. Most cases present with thrombosis of the internal jugular vein, although other veins may be involved, such as the facial vein, transverse sinus, or ophthalmic vein [1].

LS should be considered in the differential diagnosis of patients presenting with a persistent sore throat, mastoiditis, or recent dental procedures, accompanied by neck pain and swelling [7]. Management consists of appropriate antibiotic therapy combined with surgical drainage of the infected site(s) [4]. Anticoagulation, although still debated, can be considered in select cases [6].

We present a case of LS complicated by descending mediastinitis. This case highlights the importance of early, antibiotic-based management to prevent potentially fatal complications in young patients.

## Case presentation

A 35-year-old woman had been using weight-gain-promoting products, possibly corticosteroid-based, for the past three months. She presented with neck swelling, odynophagia, and fever for approximately two weeks and subsequently went to the emergency room.

On examination, she was conscious. Her vital signs were heart rate 119 beats/min, blood pressure 137/78 mmHg, and body temperature 38.5°C. Her blood oxygen saturation on room air was 93%. Chest auscultation revealed a right pleural effusion. Laboratory results at admission are summarized in Table 1. Peripheral blood analysis showed a white blood cell count of 23,100/mm³, predominantly neutrophils. C-reactive protein was markedly elevated at 260 mg/L, and D-dimer levels were also increased.

**Table 1:** Laboratory results on admission and at multiple follow-up time points after treatment

Laboratory test	At admission	Day 5	Six weeks	Reference range
White blood cells (/mm³)	23,100	12,220	7,490	4,000-10,000
Neutrophils (/mm³)	19,289	9,830	4,110	2,000-7,500
Lymphocytes (/mm³)	2,402	1,560	-	1,500-4,000
Hemoglobin (g/dL)	11.2	8.9	15.89	11.5-17.5
Platelets (/µL)	359,000	245,000	417,000	150,000-445,000
C-reactive protein (mg/dL)	260	56.3	0.9	<0.6
Prothrombin time (%)	72	70	70	70-100
Activated partial thromboplastin time	1	1	1	<1.20
D-dimer (ng/mL)	1,200	500	300	>500
Macroscopic appearance of pleural fluid	Purulent	-	-	-
Total protein of pleural fluid (g/L)	50	-	-	20
Total white blood cells of pleural fluid (/mm³)	192,000	-	-	<1,000
Predominant cell type: neutrophils of pleural fluid (%)	90.1	-	-	-
Direct examination (Gram stain) of pleural fluid	Gram-negative bacteria	-	-	-

**Figure 1:**
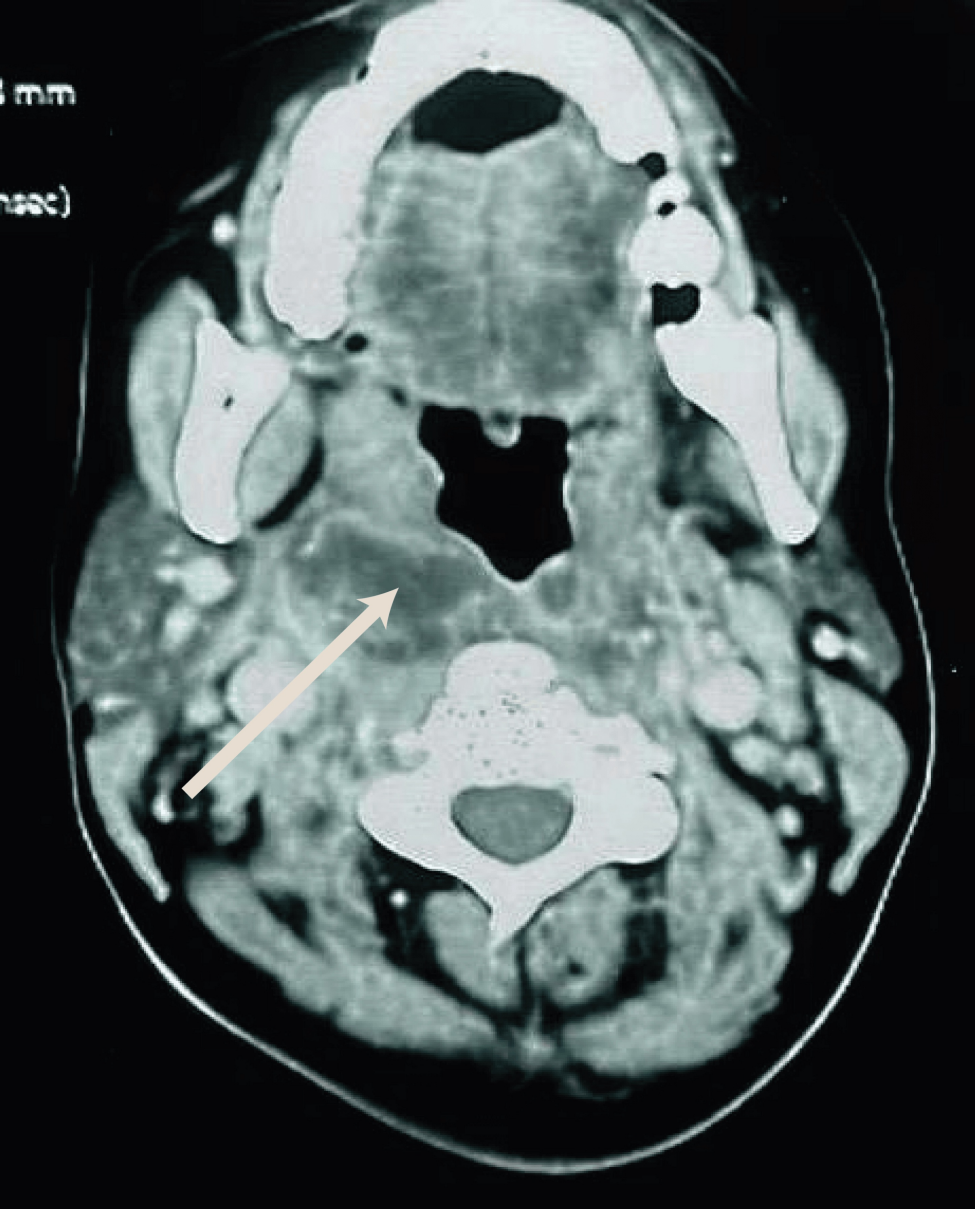
Cervical CT, axial view, parenchymal window, showing abscess collections in the retro- and parapharyngeal spaces

**Figure 2:**
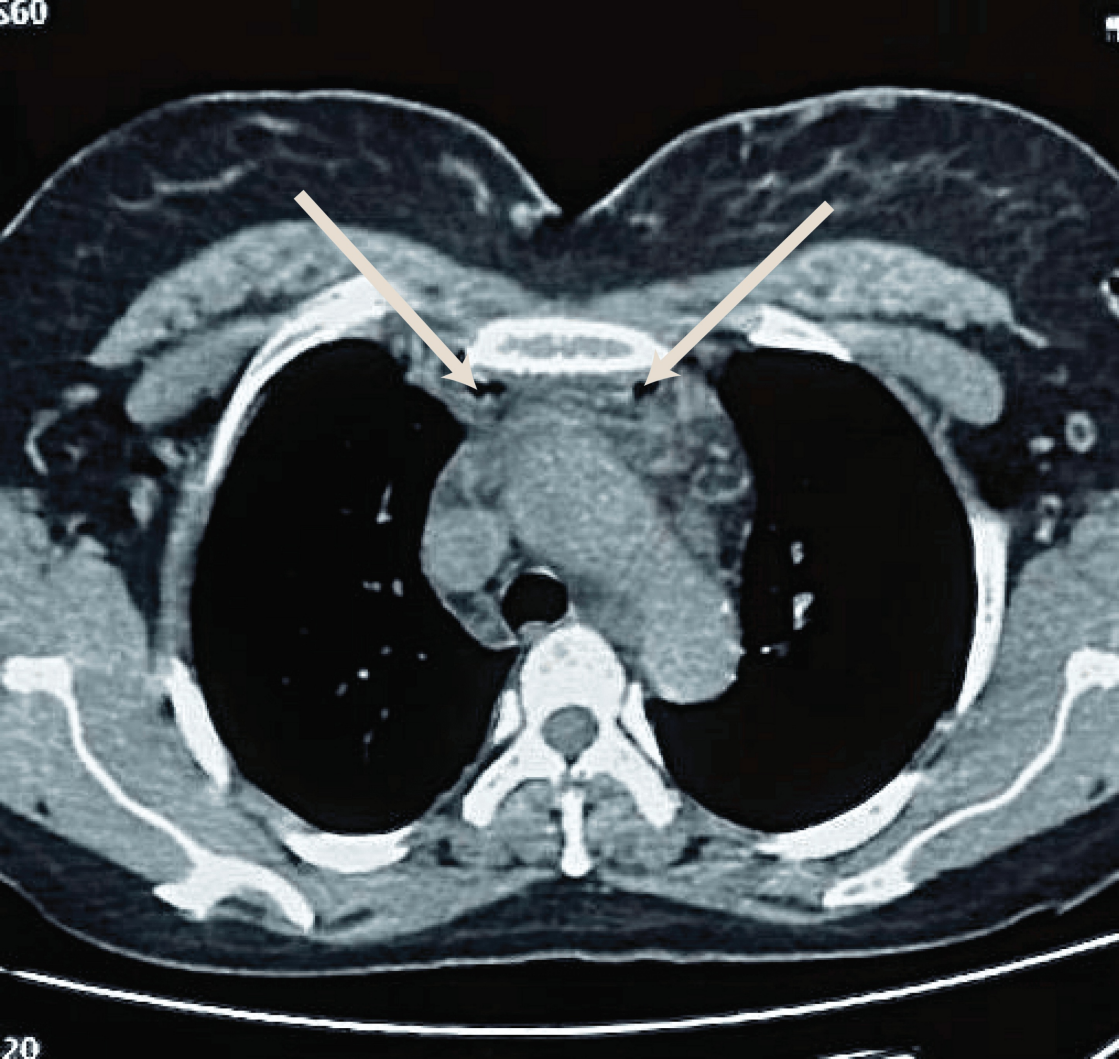
Axial thoracic CT demonstrating a hypodense collection with air bubbles in the anterior mediastinum

**Figure 3:**
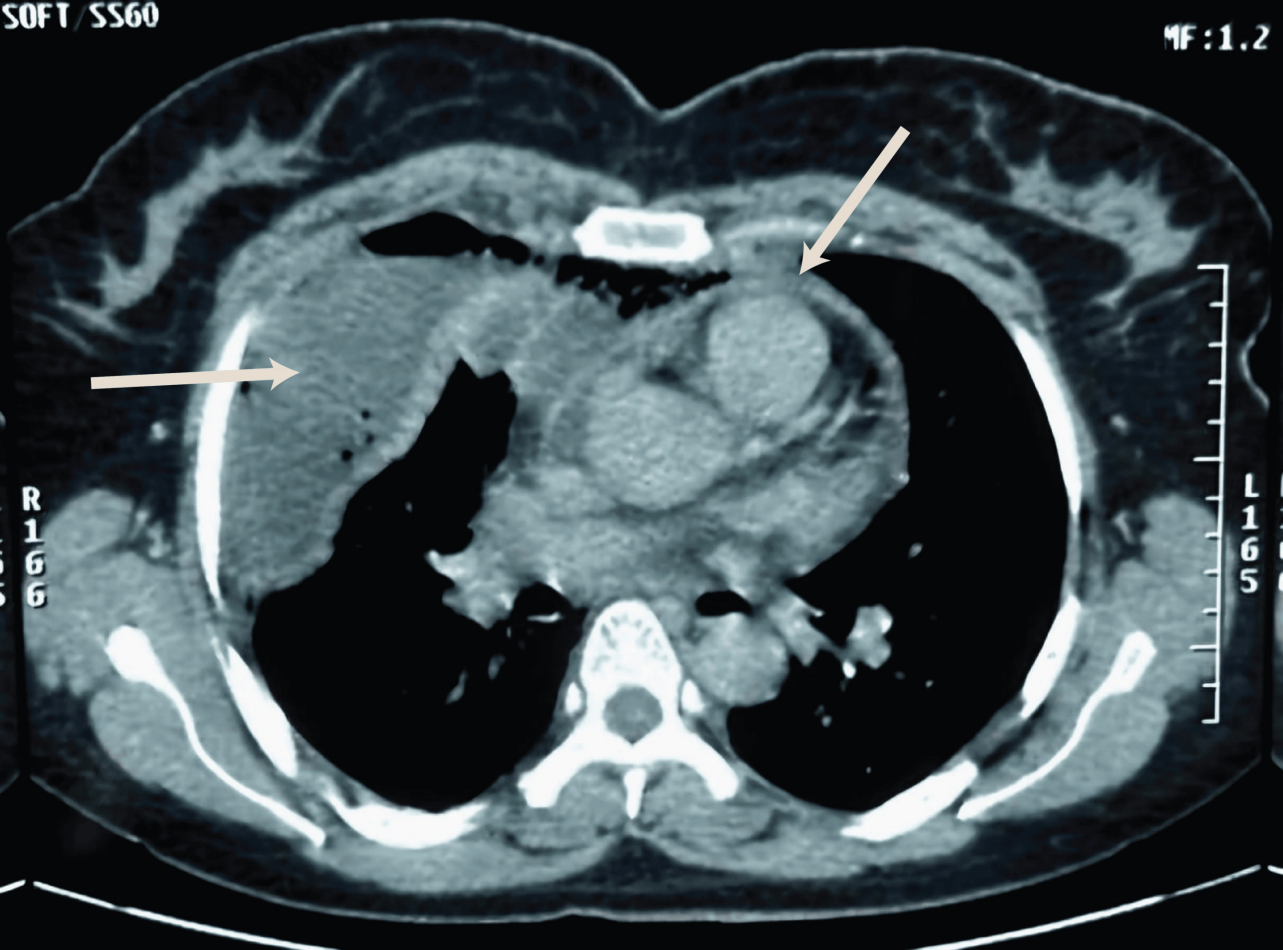
Axial thoracic CT, mediastinal window, showing right pleural empyema and a thin pericardial effusion The right arrow indicates the pleural empyema, and the left arrow indicates the pericardial effusion.

Figure 1, Figure 2, and Figure 3 illustrate the imaging findings at admission. Contrast-enhanced CT revealed laterocervical, parapharyngeal, and tonsillar abscesses extending to the anterior cervical region with air bubbles, retrosternal collections, and right pericardial and pleural effusions, as well as thrombosis of the superior vena cava, the right retromandibular vein, and the right external jugular vein.

After admission, samples were obtained from the pleural effusion and cervical collection. The patient had already received prior antibiotic therapy with amoxicillin before hospital admission. Probabilistic hospital-based treatment was initiated with ceftriaxone 2 g/day, an aminoglycoside 120 mg/day, and metronidazole 500 mg three times daily. In addition, low-molecular-weight heparin was administered at a dose of 4,000 IU twice daily.

Regarding the pericardial effusion, a transthoracic echocardiogram was performed by a cardiologist to rule out infective endocarditis. The cardiology team recommended starting the patient on aspirin and colchicine, resulting in good clinical progress and regression of the pericardial effusion. The diagnosis of LS was established based on imaging findings.

Emergency surgical treatment was performed collaboratively by the ENT and thoracic surgery teams, including drainage of the cervical abscesses, debridement of the anterior mediastinal collection, and right chest drainage. The pleural effusion was exudative, with a predominance of neutrophils (91.1%), and the bacterial culture of the fluid was negative. Inflammation improved promptly after surgical intervention and initiation of antibiotic therapy (Table 1).

**Figure 4:**
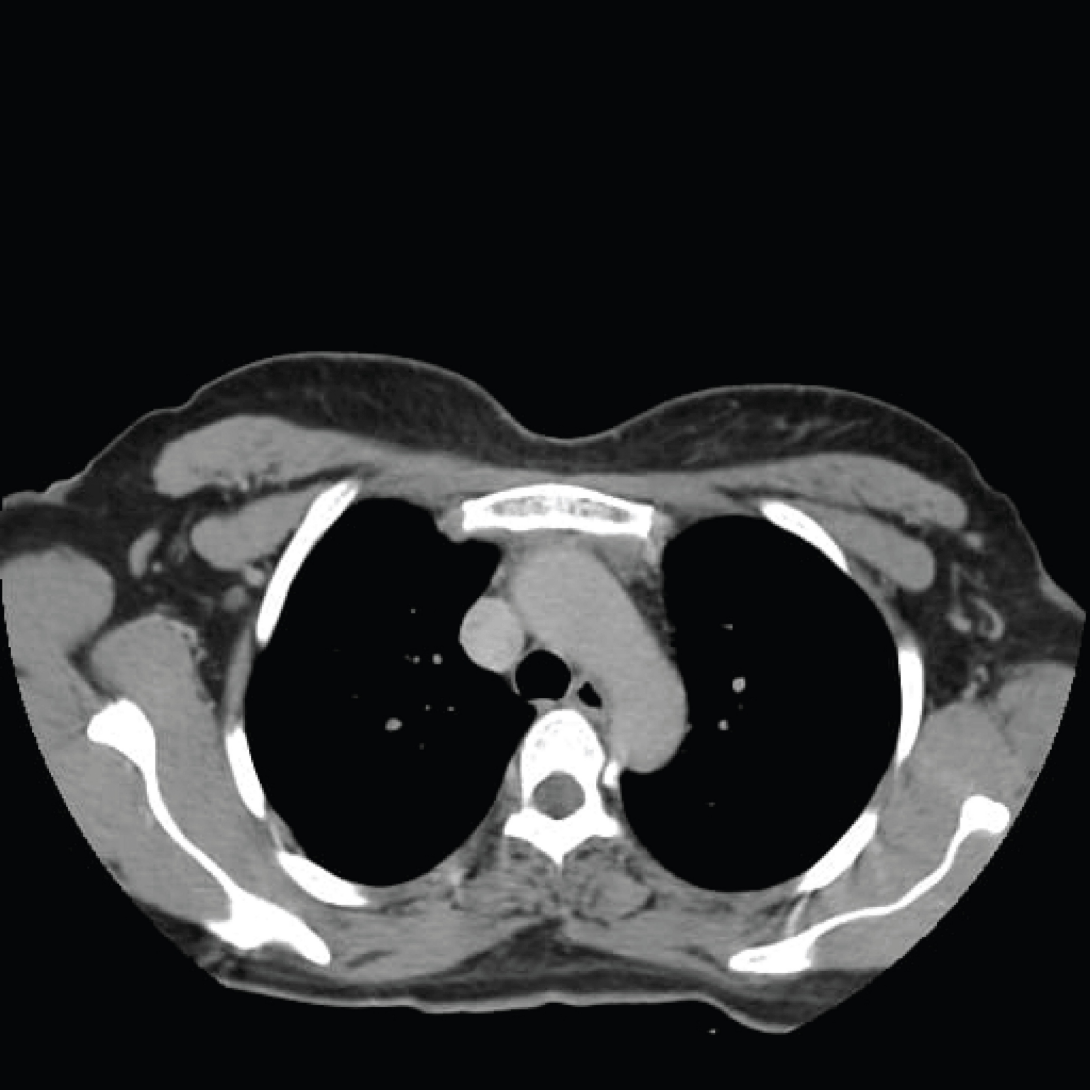
Follow-up axial thoracic CT scan showing complete resolution of the previously observed anterior mediastinal collection

**Figure 5:**
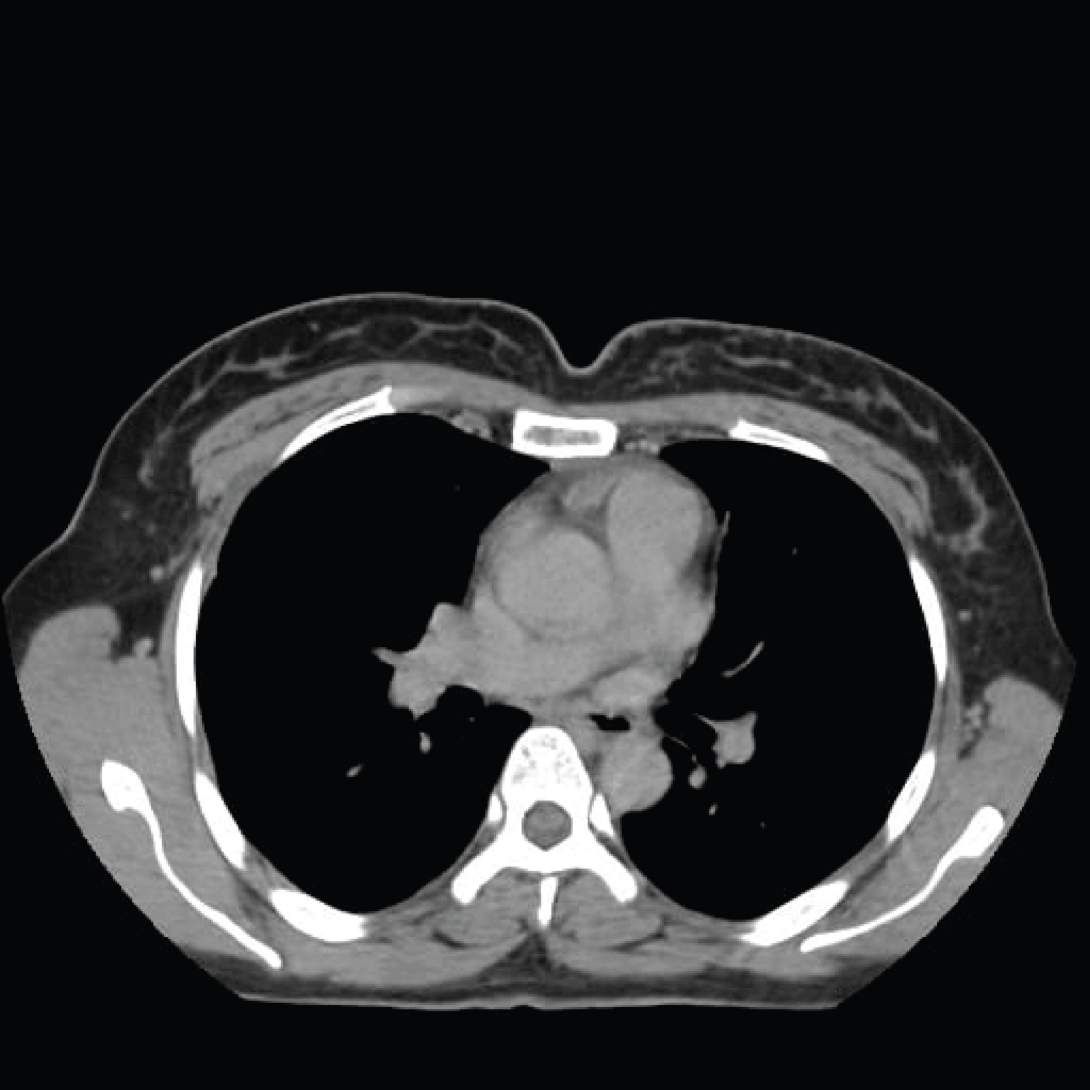
Follow-up axial thoracic CT scan in the mediastinal window demonstrating no evidence of pleural empyema or pericardial effusion

A contrast-enhanced CT scan performed on day 20 of admission confirmed resolution of thrombosis in the right external jugular vein and superior vena cava. The patient was discharged on postoperative day 21 without complications. Antimicrobial and anticoagulant therapy was discontinued on day 37 of admission. She returned to the hospital on day 42 for follow-up, with no recurrence of infection, and the treatment course was completed. Follow-up CT imaging demonstrated no residual pleural or pericardial effusion, and debridement of the anterior mediastinum and cervical region had been successfully achieved (Figure 4, Figure 5). The patient remains under ongoing follow-up.

## Discussion

LS is a rare but debilitating infection characterized by septic thrombophlebitis of the internal jugular vein following an oropharyngeal infection, with *F. necrophorum *being the most common pathogen. The incidence of LS is low, estimated at three to six cases per million population annually, with a peak incidence of 14.4 cases per million among individuals aged 14-24 years. Although its incidence has dramatically decreased in the antibiotic era, LS remains potentially fatal when diagnosis and treatment are delayed [1,8].

In the reviewed data,* F. necrophorum *remained the leading pathogen among the 96 cases in which a microbiological agent was identified. However, an additional 40 cases showed no detectable organism. This lack of microbiological confirmation may be related to prior antibiotic administration, delayed sampling, or the well-known difficulty of culturing obligate anaerobes such as fusobacteria [8].

The primary oropharyngeal infection typically begins in the tonsils or peritonsillar space, from where it may spread into the parapharyngeal and carotid spaces. Thrombophlebitis allows the dissemination of septic emboli to distant organs such as the lungs, liver, or joints [2,6]. Less commonly, the infection can spread inferiorly through the deep cervical fascial planes to the mediastinum, resulting in descending necrotizing mediastinitis (DNM) [9]. Gravity, negative intrathoracic pressure, and continuity of the cervical fascial spaces with the superior mediastinum facilitate this downward spread [5,9].

The coexistence of LS and DNM is rare and potentially life-threatening, as reported by Yang et al. [9]. Our case demonstrates that LS can be complicated by extensive venous thrombosis and mediastinal extension with pleural and pericardial involvement. Simultaneous thrombosis of multiple venous axes, including the superior vena cava, retromandibular vein, and external jugular vein, as observed in our patient, is unusual and seldom reported [4,10].

Early imaging is essential for diagnosis. Contrast-enhanced CT remains the gold standard for detecting cervical abscesses, venous thrombosis, and mediastinal spread [1,4]. In our patient, cervicothoracic CT was critical for confirming the diagnosis and characterizing the extent of infection. This underscores the need for comprehensive imaging when LS is suspected, particularly in patients with dyspnea, chest pain, or pleural effusion.

Antibiotics remain the mainstay of therapy. Empiric treatment should provide broad-spectrum coverage of anaerobic bacteria, particularly *F. necrophorum*, as well as *Streptococcus *and *S. aureus *[6,8]. First-line empiric therapy often includes a β-lactam/β-lactamase inhibitor combination or a third-generation cephalosporin with metronidazole [7,10]. Our patient received early combination therapy with ceftriaxone, aminoglycosides, and metronidazole, resulting in a favorable response, supporting the effectiveness of early synergistic antimicrobial therapy.

The role of anticoagulation in LS remains controversial. Some authors recommend it for extensive venous thrombosis, particularly when the internal jugular vein or major thoracic veins are involved, to prevent extension and embolization [6,9]. In a meta-analysis by Gore, anticoagulation was administered in approximately one-quarter of reported LS cases; however, its therapeutic benefit remains debated and should be individualized [11]. In our patient, administration of low-molecular-weight heparin was associated with positive outcomes, with no further thrombosis, consistent with recent reports suggesting improved recanalization and reduced septic complications with anticoagulation [4,6].

Mediastinal abscesses or mediastinitis require surgical management. Early drainage of cervical and mediastinal collections, along with pleural aspiration, is lifesaving and should be performed promptly by a multidisciplinary team comprising ENT and thoracic surgeons [5,9]. Our patient’s outcome illustrates the importance of multidisciplinary care involving early surgical intervention, appropriate antibiotics, and supportive measures.

The prognosis of LS has improved significantly, with current mortality rates of 2-4% for uncomplicated cases [4]. However, when accompanied by DNM or sepsis, mortality may increase substantially [5,9]. Therefore, in young patients presenting with prolonged sore throat, persistent neck swelling, and systemic sepsis unresponsive to primary therapy, clinicians should maintain a high index of suspicion for LS.

## Conclusions

LS remains an uncommon but serious complication following oropharyngeal infections and can rapidly progress to life-threatening conditions such as descending mediastinitis. Early recognition is critical, as the clinical presentation may be nonspecific and easily confused with more common bacterial infections of the pharynx. Timely diagnosis with comprehensive radiological evaluation, particularly contrast-enhanced CT, combined with aggressive therapy, including broad-spectrum antibiotics, anticoagulation when indicated, and prompt surgical drainage, is essential for improving survival. This case underscores the importance of maintaining a high index of suspicion for LS in young patients presenting with persistent neck swelling and systemic signs of infection, highlighting early intervention as key to preventing fatal complications and reducing mortality.
